# A Mitogenomic Re-Evaluation of the Bdelloid Phylogeny and Relationships among the Syndermata

**DOI:** 10.1371/journal.pone.0043554

**Published:** 2012-08-23

**Authors:** Erica Lasek-Nesselquist

**Affiliations:** University of Connecticut, Department of Molecular and Cellular Biology, Storrs Connecticut, United States of America; Laboratoire Arago, France

## Abstract

Molecular and morphological data regarding the relationships among the three classes of Rotifera (Bdelloidea, Seisonidea, and Monogononta) and the phylum Acanthocephala are inconclusive. In particular, Bdelloidea lacks molecular-based phylogenetic appraisal. I obtained coding sequences from the mitochondrial genomes of twelve bdelloids and two monogononts to explore the molecular phylogeny of Bdelloidea and provide insight into the relationships among lineages of Syndermata (Rotifera + Acanthocephala). With additional sequences taken from previously published mitochondrial genomes, the total dataset included nine species of bdelloids, three species of monogononts, and two species of acanthocephalans. A supermatrix of these 10–12 mitochondrial proteins consistently recovered a bdelloid phylogeny that questions the validity of a generally accepted classification scheme despite different methods of inference and various parameter adjustments. Specifically, results showed that neither the family Philodinidae nor the order Philodinida are monophyletic as currently defined. The application of a similar analytical strategy to assess syndermate relationships recovered either a tree with Bdelloidea and Monogononta as sister taxa (Eurotatoria) or Bdelloidea and Acanthocephala as sister taxa (Lemniscea). Both outgroup choice and method of inference affected the topological outcome emphasizing the need for sequences from more closely related outgroups and more sophisticated methods of analysis that can account for the complexity of the data.

## Introduction

Rotifera and Acanthocephala (Syndermata) persist as phylogenetically problematic invertebrate taxa despite numerous attempts to decipher their evolutionary history. Here, I present the first mitogenomic exploration of relationships within the rotifer class Bdelloidea in an attempt to resolve one piece of the puzzle. Additionally, I evaluate how increased taxonomic-breadth of sampling, outgroup selection, and different methods of inference alter the phylogeny of Syndermata and compare these results to previous mitogenomic analyses. The phylum Rotifera traditionally includes three classes: Monogononta, Bdelloidea, and Seisonidea, representing cyclical parthenogens, apparently strict parthenogens, and obligate bisexuals, respectively. Monogononta and Bdelloidea are free-living and found in aquatic or ephemerally aquatic environments [1]. While there are hundreds of monogonont and bdelloid species [2], Seisonidea consists of only three described species, each of which forms an ecto-symbiotic/commensal relationship with marine crustaceans of the genus *Nebalia* [3], [4]. Molecular and morphological support exist for including the sexual, endoparasitic phylum, Acanthocephala within Rotifera as well but whether Acanthocephala falls sister to Bdelloidea, Monogononta, or Seisonidea remains uncertain [5].

Bdelloidea consists of four families - Habrotrochidae, Philodinidae, Adinetidae, and Philodinavidae – and essentially retains a morphological classification system that is over 80 years old [6], [7]. Under the traditional classification system, Philodinidae and Habrotrochidae belong to the same order, Philodinida, sharing similar organization of the corona (ciliated wheel organ) and rostrum (an adhesive structure that aids in creeping movement with the foot) [6], [7]. Similarly, a most parsimonious tree derived from 60 morphological, embryological, and biochemical characters revealed a monophyletic Philodinida with Adinetidae as sister to this group [8]. However, molecular analyses of the bdelloid phylogeny, which could contribute to proper identification and classification of these small invertebrates, are severely lacking [9], [10].

I sought clarification of the bdelloid phylogeny and specifically tested the evidence for monophyletic relationships among bdelloid families as well as sister relationships among genera by analyzing a supermatrix of 10–12 mitochondrial coding sequences from 13 bdelloids (representing 8 species and three out of four bdelloid families), three monogononts, and two acanthocephalans. Animal mitochondrial genomes typically consist of 12–13 coding sequences - *atp6*, *cox1*, *cox2*, *cox3*, *cytb*, *nd1*, *nd2*, *nd3*, *nd4*, *nd4l*, *nd5*, *nd6*, and sometimes *nd8*, a large and small ribosomal subunit (*lsu* and *ssu*), and 22 tRNAs – usually found as a single circular molecule [11]. Mitochondrial genes are maternally inherited as a single unit and as such, usually reflect the same evolutionary history [12]. Because bdelloids are degenerate tetraploids [13], [14], the potential exists for incorporating paralogous nuclear sequences into a dataset and generating trees that reflect a chimeric history. An analysis of mitochondrial genomes circumvents this issue.

In addition to avoiding the problems of paralogy, I performed several different phylogenetic analyses on my mitochondrial dataset in an attempt to overcome any inherent biases. Several factors could contribute to model violations and result in long-branch attraction (LBA; the artificial attraction of two rapidly evolving or compositionally similar taxa that are not each other’s closest relatives [15], including taxonomic sampling, outgroup choice, rate variation across sites, and compositional heterogeneity [16], [17], [18]. I attempted to address these issues by increasing the taxonomic sampling of Bdelloidea, selecting closely related outgroups (Monogononta and Acanthocephala), choosing appropriate models of amino acid substitution, and accounting for rate and compositional heterogeneity. My results provide little justification for maintaining the order Philodinida or the family Philodinidae and emphasize the need for increased taxonomic sampling, more phylogenetically informative data, and better models of protein evolution to resolve the bdelloid phylogeny.

Finally, I rooted Syndermata with Platyhelminthes and Chaetognatha to recover relationships among Acanthocephala, Monogononta, and Bdelloidea. Because it is difficult to assess how these relationships might change with the introduction of sequences from Seisonidea, I compared my results with two recent mitogenomic analyses and a study that included >1000 *cox1* sequences from the same syndermate lineages [9], [19], [20]. Both previous mitogenomic analyses support a sister relationship between Acanthocephala and Bdelloidea (Lemniscea) [19], [20]. The results from the large-scale *cox1* study are inconclusive: topology tests support Lemniscea but the best maximum likelihood and Bayesian trees support a sister relationship between Bdelloidea + Monogononta (Eurotatoria) [9]. I incorporated the two strategies employed by these three studies – more sequences and greater taxonomic representation – in attempt to improve resolution. My analyses reveal that Acanthocephala consistently falls sister to Eurotatoria when site-homogeneous, empirical amino acid substitution models are applied to a syndermate dataset rooted by Platyhelminthes. With a more complex model and/or a more slowly evolving outgroup (Chaetognatha), a sister relationship between Acanthocephala and Bdelloidea emerges. Relationships among subtaxa of Syndermata appear rather unstable and highly influenced by outgroup selection. Thus, care should be taken to reduce the effects of systematic biases.

## Materials and Methods

### Rotifer Culture and Molecular Techniques

Thirteen bdelloids, three monogononts, and two acanthocephalans represented Syndermata in the present analyses (Table 1). Members of Bdelloidea include: *Adineta vaga* and *Adineta ricciae* representing the family Adinetidae, *Macrotrachela quadricornifera*, *Philodina roseola*, *Philodina acuticornis*, and *Rotaria rotatoria* representing the family Philodinidae, and *Habrotrocha rosa* and *Habrotrocha constricta* representing the family Habrotrochidae (Table 1). *Brachionus plicatilis*, *Brachionus manjavacas*, and *Brachionus calyciflorus* represented Monogononta (Table 1). Cultures of *Adineta vaga*, *Habrotrocha constricta*, and six distinct but closely related clonal cultures of *Macrotrachela quadricornifera* (“MM”, “MA”, “CR”, “LH”, “HR”, and “MQ” obtained from M. Meselson) grew in filtered or autoclaved Poland Spring water and subsisted on *E. coli* strain M28. I extracted DNA using a bead-beater and 0.1 mm silica beads to break open the rotifers, followed by lysis in an SDS/proteinase K (20 mg/ml) solution and a standard phenol chloroform extraction with ethanol/sodium acetate precipitation. Degenerate primers from Min and Park (2009) initially amplified small fragments of the *cox1*, *cox2*, and *16s* mitochondrial genes from *M. quadricornifera*, *A. vaga*, and *H. constricta* [20]. The sequences generated from these amplicons then directed the design of primers specific to these taxa. The NEB LongAmp PCR kit (NEB, Ipswich, MA) generated 5–12 Kb amplicons. Either the hydroshear (GeneMachines, San Carlos, CA, USA) or the Covaris S2 (Covaris Inc, Woburn, MA, USA) sheared PCR products into 500–1500 Kb pieces. T4 DNA polymerase (Invitrogen, Carlsbad, CA) end-repair, dephosphorylation of 5′ ends with Antarctic phosphatase (NEB, Ipswich, MA, USA), and 5′ A tail addition using GoTaq polymerase (Promega, Madison, WI, USA) proceeded DNA shearing. Qiagen PCR Qiaquick purification columns removed enzymes after each reaction (Qiagen, Valencia, CA, USA). Amplicons were then ligated in to the Topo TA 2.1 PCR or Topo TA 4.0 sequencing vector and transformed into TOP10 electrocompetent cells (Invitrogen, Carlsbad, CA, USA). Colonies were grown in 96-well plates and the Biomek FX liquid handling robot (Beckman Coulter, Fullerton, CA, USA) purified positive plasmids using a standard alkaline lysis protocol. The Applied Biosystem 3730 XL capillary sequencer (Applied Biosystems, Foster City, CA, USA) performed all sequencing, which included both forward and reverse reads generated with M13 primers and ABI BigDye 3.1 chemistry.

**Table 1:** Species, taxonomic classification, and sequencing coverage of mitochondrial genes.

Species	Phylum/Class/Family	Missing genes	AA sequence length
*Macrotrachela quadricornifera*	Rotifera/Bdelloidea/Philodinidae*	None	3361
*Habrotrocha constricta*	Rotifera/Bdelloidea/Habrotrochidae*	*nd2*	3341
*Habrotrocha rosa*	Rotifera/Bdelloidea/Habrotrochidae*	None	3257
*Philodina roseola*	Rotifera/Bdelloidea/Philodinidae*	Truncated *cox1* & *nd5*	3062
*Philodina acuticornis*	Rotifera/Bdelloidea/Philodinidae*	Truncated *cox1* & *nd6*	2952
*Rotaria rotatoria*	Rotifera/Bdelloidea/Philodinidae	None	3340
*Adineta ricciae*	Rotifera/Bdelloidea/Adinetidae*	None	3305
*Adineta vaga*	Rotifera/Bdelloidea/Adinetidae*	None	3348
*Brachionus plicatilis*	Rotifera/Monogononta/Brachionidae	None	3413
*Brachionus manjavacas*	Rotifera/Monogononta/Brachionidae*	*nd6*	3160
*Brachionus calyciflorus*	Rotifera/Monogononta/Brachionidae*	*nd2*, *nd3*	2698
*Leptorhynchoides thecatus*	Acanthocephala/Palaeacanthocephala/Rhadinorhynchidae	None	3410
*Oncicola luehei*	Acanthocephala/Archiacanthocephala/Oligacanthorhynchoidae	None	3457
*Benedenia hoshinai*	Platyhelminthes/Monogenea/Capsalidae	None	3289
*Schistosoma mansoni*	Platyhelminthes/Trematoda/Schistosomatidae	None	3306
*Paraspadella gotoi*	Chaetognatha/Phragmorpha/Spadellidae	*atp6*	3201
*Spadella cephaloptera*	Chaetognatha/Phragmorpha/Spadellidae	*atp6*	3206

“None” indicates the presence of a complete mitochondrial coding sequence repertoire (*atp6*, *cox1-cox3*, *cytb*, *nd1-nd6*, and *nd4l*).

* Mitochondrial sequences generated from this study; AA sequence length, total length of concatenated amino acid sequences from mitochondrial genes. The mitochondrial genomes of all six clonal cultures of *Macrotrachela quadricornifera* were sequenced completely. The gene, *atp6* is not present in Chaetognatha.

Total RNA was isolated from clonal cultures of *A. ricciae*, *P. acuticornis*, *P. roseola*, *H. rosa*, and *B. manjavacas* using RNAqueous Micro Kit (Ambion). Synthesis of cDNA with first strand primer 5′ CTA GAG GCC GAG GCG GCC GAT TTT TTT TTT TTT TTT TTT UVN 3′ made use of the template switching property of Superscript II (Invitrogen) to incorporate barcoded, biotintylated 5′ adapters that matched the “A” sequence primers used in 454 FLX pyrosequencing (5′ GCC TCC CTC GCG CCA TCA Gxx xxx GG, where xxxxx is CACTG for *B. manjavacas*). To prepare libraries for pyrosequencing, 3–5 µg of cDNA was sheared using an Aeromist Nebulizer (Allied Healthcare Products) for 3–4 min at 50 psi N_2_, and the biotintylated 5′ EST ends of the fragmented library were captured with Dynabeads M-280 Streptavidin (Invitrogen). The captured DNA was end repaired using a Quick Blunt Kit (New England BioLabs) and ligated to a modified 454 FLX “B” adapter (AAG CCT TGC CAG CCC GCT CAG T) following A-tailing with Taq polymerase. Libraries were quantified and sequenced on a Roche GS FLX using the manufacturers protocols. Resting eggs of *Brachionus calyciflorus* Florida strain (Florida Aqua Farms Inc., USA) were hatched in MBL medium. Neonates were collected, washed with sterile DI water, and pelleted by centrifugation. DNA was extracted using the DNeasy Blood and Tissue Kit (Qiagen, Valencia, CA) according to the manufacturer’s instructions, and purified using the Microcon Centrifugal Filter device YM-100 (Millipore, Billerica, MA). A genomic DNA library was prepared using the Nextera DNA Sample Prep Kit (Epicentre, Madison, WI) and sequenced using the Roche 454 GS-FLX with Titanium chemistry.

Sequences were trimmed of the 5′ barcode and 3′ cDNA primer (if present) using in-house Perl scripts, and assembled using Newbler v.2.5 [21]. TBLASTX similarity searches [22] of these transcriptomic and genomic libraries against an MA mitochondrial reference identified putative mitochondrial coding sequences.

### Sequence Editing and Genome Assembly

For *M. quadricornifera*, *A. vaga*, and *H. constricta*, Phred v.1.08 called bases and assigned quality values, crossmatch v.108 removed all vector sequence, and an in-house Perl script assembled forward and reverse reads together when present [23], [24]. Either the genome assembler Phrap v.1.08 with default settings or Mira v.3.4, with a reference sequence option assembled reads into contigs [23], [24], [25]. If necessary, site specific PCRs mediated gap closure. Independent PCRs verified polymorphisms among *M. quadricornifera*, *A. vaga*, and *H. constricta* genomes. Assemblies were visualized and edited in Consed v.19 [26].

### Additional mtDNA Sequences

The acanthocephalan mt genomes from *Leptorhynchoides thecatus* (GenBank # NC_006892; [27] and *Oncicola luehei* (GenBank #NC_016754; [19] the monogonont mt genome of *Brachionus plicatilis* (GenBank #s NC_010472 and NC_010484; [28], and a previously sequenced bdelloid genome from *Rotaria rotatoria* (GenBank #NC_013568; [20] were downloaded from GenBank and included in phylogenetic analyses (Table 1). Outgroup sequences derived from the Platyhelminthes *Benedenia hoshinai* and *Schistosoma mansoni* (GenBank #s NC_014591 and NC_002545) and the Chaetognatha *Spadella cephaloptera* and *Paraspadella gotoi* (GenBank #s AY545549 and AY619710; Table 1). Mitochondrial sequences generated or mined in this study were submitted to GenBank and given the accession numbers: JX183989–JX184083 and JW861079–JW861112.

### Mitochondrial Sequence Annotation and Alignment Properties

GLIMMER v.3.0 [29] identified open reading frames while BLAST similarity searches using TBLASTX [22] against *R. rotatoria* coding sequences distinguished legitimate and non-legitimate ORF calls. Muscle v.3.8.31 [30] generated amino acid alignments and MEGA v5.0 [31] generated in-frame nucleotide alignments for individual mitochondrial genes. The CLC DNA workbench (http://www.clcbio.com) or an in-house Python script concatenated gene alignments into a supermatrix. MEGA also provided summary statistics such as GC and amino acid compositions for coding sequences and the relative rate test for estimating different rates of evolution among taxa [32]. TREE-PUZZLE v.5.2 [33] provided estimates of compositional homogeneity among sequences under JTT, MtREV, and WAG models of amino acid substitution with site rate variation modeled by a gamma distribution.

### Phylogenetic Analyses

I employed Bayesian and maximum likelihood (ML) analyses to reconstruct the relationships among lineages of Bdelloidea and Syndermata and a variety of strategies to reduce compositional biases, decrease the effects of saturation, and assess the reliability of my results (summarized in Table 2). These strategies included applying different models of amino acid evolution with and without data partitioning, choosing different outgroups, and recoding amino acid alignments into Dayhoff categories [34]. Dayhoff recoding helps mitigate the effects of compositional heterogeneity by binning amino acids into 6 groups (AGPST, C, DENQ, FWY, HKR, ILMV) based on biochemical properties, such as being a positively charged amino acid [18], [34]. These bins reflect amino acids that tend to replace each other [18], [34].

**Table 2:** Support for alternative bdelloid roots and major bdelloid and syndermate clades from various phylogenetic analyses.

	Bdelloid phylogenies							Syndermate phylogenies		
Strategy	P, others	M, others	RHM	HAP	PRA	HRA	lnL	Eurotatoria	Lemniscea	lnL
ML + JTT		38		20			−49614	59	**98**	−63252, −**61722**
ML + MtRev	32		13				−49731	51	**97**	−63446, −**61877**
ML + Partitioned	41		16				−49428	58	**96**	−63145, −**61670**
ML + JTT recoded	78		60				−23983	86	**82**	−31552, −**30021**
MB + Mixed (MtRev)		0.88			0.57		−53759	0.98	**1.0**	−68553, −**64585**
MB + WAG	1.0		0.98				−54287			
PB + CAT		0.67				0.75	−42526		0.57, **0.99**	−56324, −**54400**
PB + CAT + GTR	0.5		0.5				−44997			
MP	100		100							

Strategy indicates 1) method of inference (ML, maximum likelihood; MB, Bayesian inference in MrBayes; PB, Bayesian inference in Phylobayes; MP, maximum parsimony), 2) model of substitution (JTT, MtRev, Mixed, and WAG, empirical amino acid models; CAT, site heterogeneous model of Phylobayes with Poisson or GTR exchange profiles), 3) and other data manipulation techniques (partitioned, alignment partitioned by proteins sharing the same model of evolution; recoded, alignments Dayhoff-recoded). The bootstrap support values and posterior probabilities for bdelloid roots as well as bdelloid and syndermate major clades recovered from these analyses are provided (P, others − bdelloid root between *Philodina* and others; M, others − bdelloid root between *Macrotrachela* and others; RHM − *Rotaria*, *Habrotrocha*, *Macrotrachela* clade; HAP – *Habrotrocha*, *Adineta*, *Philodina* clade; PRA − *Philodina*, *Rotaria*, *Adineta* clade; HRA − *Habrotrocha*, *Rotaria*, *Adineta* clade; Eurotatoria, support for Bdelloidea + Monogononta; Lemniscea, support for Bdelloidea + Acanthocephala. There are two values listed for each syndermate analysis, regular type indicates results from the Syndermata + Platyhelminthes dataset and boldface indicates results from the Syndermata + Chaetognatha dataset. lnL, log-likelihoods.

RAxML v.7.0.4 or v.7.2.8 [35], [36] reconstructed maximum likelihood trees from bdelloid and syndermate amino acid alignments (Table 2). All analyses ran under a gamma distribution of among site rate-heterogeneity with four rate categories (the PROTGAMMA option) with 100–500 bootstrap replicates, a random seed, and amino acid substitution models chosen by ProtTest v.2.4 [37], [38], [39](Table S1). ML analyses reconstructed bdelloid and syndermate phylogenies from partitioned and non-partitioned data (Table 2). The model(s) assigned to the majority of the mitochondrial proteins (JTT, see Table S1) or chosen by the MrBayes MCMC sampler (see below) served as the model(s) for non-partitioned alignments (Table 2). Proteins assigned the same models of evolution were grouped into the same partition to avoid over-fitting the data, which might occur if each protein was assigned to its own partition [40]. Additionally, RAxML and the MEGA implementation of parsimony analysis generated phylogenies based on Dayhoff-recoded alignments (Table 2).

MrBayes v.3.1.2 [41] and Phylobayes v.3.2e [42] performed all Bayesian inferences (Table 2). MrBayes analyses comprised of two runs of 1 or 2 million generations sampled every 100^th^ generation with the first 200,000–250,000 generations discarded as burn-in. Bayesian analyses included an alpha-shaped gamma distribution of across site-rate heterogeneity and a proportion of invariant sites (I+G). In lieu of certain models specified by ProtTest but not available in MrBayes (such as JTT), I assigned the WAG model to the bdelloid alignment and the mixed model of evolution to bdelloid and syndermate alignments (Table 2). The mixed model of evolution allows the MCMC sampler to choose the best amino acid substitution model (MrBayes manual) – in these cases MtRev for bdelloid and syndermate phylogenies. Tracer v.1.5.0, from the BEAST package, graphically displayed the trace files from MrBayes analyses and allowed evaluation of chain mixing and run convergence and confirmed the adequacy of burn-in times [43].

Phylobayes offers an advantage over MrBayes and RAxML by applying the CAT model of amino acid evolution, which more accurately infers amino acid substitutions [42], [44]. Essentially, CAT is a site heterogeneous model that estimates site-specific equilibrium frequencies and provides a greater robustness to LBA artifacts than the site-homogeneous models offered in MrBayes and RAxML [42], [44]. Phylobayes ran with the following default parameters for bdelloid and syndermate analyses: a discrete gamma distribution of rate variation with four rate categories, relative exchange rates modeled by Poisson and or GTR (general time reversible) processes, and the CAT model to estimate amino acid profiles. Two independent chains ran until reaching convergence, which was determined by the “bpcomp” command. Bpcomp evaluates the discrepancy of bipartition frequencies between the two runs and outputs a consensus tree (Phylobayes manual). A difference in bipartition frequencies of less than 0.1 indicates adequate convergence of the two runs (Phylobayes manual); for all of my analyses, maximum differences ranged between 0.04–0.16.

I rooted Bdelloidea with sequences from the most closely related taxa available - Monogononta and Acanthocephala - to provide topological balance and decrease the chances of homoplasy [16] although Monogononta and Acanthocephala still fall on relatively long branches with respect to Bdelloidea. I chose Platyhelminthes to serve as an outgroup to Syndermata because Platyhelminthes shows a close association with Syndermata [19], [45], [46], [47], [48], [49] and mitochondrial genomes from representatives of Gnathifera (a clade comprised of Gnathostomulida and perhaps Cycliophora, Micrognathozoa, and Gastrotricha) - a probable sister group to Rotifera [5], [8], [50], [51], [52], [53] - have not been sequenced yet. Despite being the most closely related group to Syndermata available for outgroup sequences, most members of Platyhelminthes are rapidly evolving, which potentially promotes LBA to rapidly evolving members of the ingroup [46], [54]. Two members of Chaetognatha – another phylogenetically elusive invertebrate phylum - served as an alternative root to assess whether outgroup choice affected the topology of Syndermata.

### Topology Testing

The approximately unbiased (AU) test and the Shimodaira-Hasegawa (SH) test, as implemented in CONSEL v.0.1i [55] evaluated the possibilities of monophyletic Adinetidae + Philodinidae, Adinetidae + Habrotrochidae, and Philodinidae + Habrotrochidae clades as well as whether all possible sister relationships among bdelloid genera were equally plausible. RAxML reconstructed the most likely trees with these groups constrained as monophyletic and calculated the site likelihood values for these trees. I also tested all possible relationships of Acanthocephala to Monogononta and Bdelloidea to determine whether any relationships could be definitively ruled out.

**Table 3:** Results from Dayhoff-recoding alignments to reduce compositional biases.

Taxon	Original data P-values (%)	Recoded data P-values (%)
*A.vaga*	28	97
*A. ricciae*	**0.96**	94
*H. rosa*	**0.02**	83
*H. constricta*	**0.07**	64
*M. quadricornifera* (CR)	6.35	89
*M. quadricornifera* (HR)	**0.02**	90
*M. quadricornifera* (LH)	7.85	92
*M. quadricornifera* (MA)	**4.58**	90
*M. quadricornifera* (MM)	**4.94**	91
*M. quadricornifera* (MQ)	9.86	89
*P. roseola*	**0.31**	84
*P. acuticornis*	**0.5**	65
*R. rotatoria*	99	99
*B. calyciflorus*	**0**	**0**
*B. manjavacas*	**0**	**0**
*B. plicatilis*	**0**	**0.01**
*O. luehei*	**0**	**0**
*L. thecatus*	**0**	**0**
*P. gotoi*	**0**	**0**
*S. cephaloptera*	**0**	**0**
*B. hoshinai*	**0**	**0**
*S. mansoni*	**0**	**0**

Bold type indicates significantly deviating amino acid compositions.

## Results

### Bdelloid Mitochondrial Genomes

I sequenced the complete mitochondrial genomes of six *M. quadricornifera* clonal cultures (MA, MM, LH, HR, MQ, and HR), which form a monophyletic clade as well as the complete mitochondrial genome of *A. vaga* and 11/12 genes from *H. constricta* (Table 1). Average assembly coverage was 14X with an average read length of 733 bp using both forward and reverse reads. The mitochondrial genomes of *M. quadricornifera* are ∼1 Kb shorter than *R. rotatoria* and are very similar in size to each other, ranging from 14,201 bp –14,204 bp in length. The genome of *A. vaga* is the shortest at 14,032 bp. I obtained all or most mitochondrial coding sequences for *A. ricciae*, *H. rosa*, *P. roseola*, and *P. acuticornis* (Table 1) from transcriptome libraries. Because all complete bdelloid mitochondrial genomes sequenced thus far contained the same 12 coding sequences (*atp6*, *cox1-cox3*, *cytb*, *nd1-nd6*, and *nd4l*) and 2 ribosomal subunits (*ssu* and *lsu*) and shared complete synteny, I chose to focus on phylogenetic analysis of mitochondrial proteins instead of gene order or gene presence/absence. Additionally, I could not assess mitochondrial genome sizes or gene order information from transcriptomic datasets except in the instances of polycistronic sequences that contained gene order information for 2–4 genes.

### Brachionus Mitochondrial Sequences

I obtained mitochondrial sequences from *B. manjavacas* and *B. calyciflorus* transcriptomic and genomic datasets, respectively and compared these sequences to the previously published mitochondrial genome of *B. plicatilis*. I found all mitochondrial coding sequences except *nd6* for *B. manjavacas* and *nd2* and *nd3* for *B. calyciflorus* (Table 1). The contigs assembled from reads sequenced by 454 technology could not provide confirmation of synteny among monogonont mitochondrial genomes (with the exception of polycistronic contigs) nor could they establish whether *B. manjavacas* and *B. calyciflorus* divided their mitochondrial genomes between two circular chromosomes as in *B. plicatilis* [28].

### Nucleotide and Amino Acid Composition

Syndermata and outgroup sequences displayed variable intra and inter-group GC compositions (Monogononta 29–36% GC, Acanthocephala 28–41% GC, Bdelloidea 20–26% GC, Platyhelminthes 26–31% GC, and Chaetognatha 27–36% GC). The disparate GC composition in the datasets and the long evolutionary periods of history being considered recommended analysis at the protein level, where extreme compositional biases are potentially mitigated and the potential for substitution-rate saturation decreases. However, chi-square tests for compositional homogeneity indicated that the amino acid compositions for most rotifer sequences significantly differed from the frequency distributions assumed by several empirical models (JTT, MtREV, and WAG). Only *R. rotatoria*, *A. vaga,* and the six *M. quadricornifera* isolates passed the chi-square test of homogeneity with P-values of 0.05 or greater (Table 3). The failure of empirical models to adequately reflect the amino acid frequencies of monogonont, acanthocephalan, and many bdelloid mitochondrial sequences potentially undermines the validity of the trees generated with these matrices depending on the severity of the violation. While Dayhoff-recoding enabled all bdelloids to pass the chi-square test for homogeneity, monogonont and acanthocephalan sequences still failed (Table 3). Both outrgoups to Syndermata (Platyhelminthes and Chaetognatha) failed the test of compositional homogeneity whether recoded or not (Table 3).

### Phylogenetic Relationships of Bdelloidea

**Figure 1:**
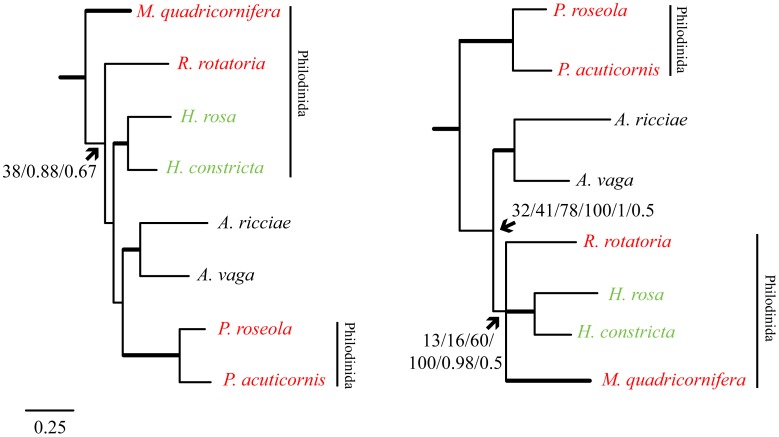
Bdelloid phylogenies. Maximum Likelihood and Bayesian phylogenies reconstructed with concatenated mitochondrial proteins and rooted with Monogononta and Acanthocephala. Left and right trees represent two predominant topologies, where the root lies between *Macrotrachela* and the rest of the bdelloids (left) or *Philodina* and the rest of the bdelloids (right). The left tree represents the topology recovered from three analyses: 1) ML + JTT, 2) MrBayes + MtRev, and 3) Phylobayes with CAT + Poisson model. Bootstrap support values and posterior probabilities for the position of the root are listed in the order given above for each analysis. The right tree represents the topology recovered from six analyses: 1) ML + MtRev, 2) ML + Partitioned data (models were assigned to each partition and partitions were generated by grouping together genes that shared the same model) 3) ML + JTT on Dayhoff-recoded data, 4) Maximum Parsimony, 5) MrBayes + WAG, and 6) Phylobayes with CAT + GTR model. Bootstrap support values and posterior probabilities for the position of the root and the RHM clade (*Rotaria*, *Habrotrocha*, and *Macrotrachela*) are listed in the order given above for each analysis. Thicker branches indicate clades strongly supported by most analyses (bootstrap support or posterior probability >90). Red taxon labels indicate members of the family Philodinidae, green taxon labels indicate the family Habrotrochidae, and black indicates the family Adinetidae. Neither Philodinidae nor the order Philodinida is monophyletic. Line represents 0.25 amino acid substitutions per site. Summaries of strategies and support value provided in Table 2.

No strategy employed recovered a monophyletic order Philodinida or monophyletic family Philodinidae due specifically to the fact that *Macrotrachela* and *Philodina* never formed a monophyletic association (Fig. 1). Additionally, *Habrotrocha* frequently broke up the possible monophyletic relationship among the remaining Philodinidae by falling sister to *Rotaria* or *Macrotrachela* instead of *Rotaria* and *Macrotrachela* branching as each other’s closest relatives.

For all ML analyses except one, *Philodina* emerged as the earliest diverging bdelloid lineage (Fig. 1; Table 2). When *Philodina* emerged at the root of the bdelloid tree, *Adineta* fell sister to a clade comprised of Habrotrochidae (*Habrotrocha*) and the remaining Philodinidae (*Rotaria* and *Macrotrachela*) but the relationships within this RHM clade varied (Fig. 1; Table 2). The application of the JTT model recovered *Macrotrachela* at the root of the bdelloid tree instead (Fig. 1; Table 2). Bootstrap support values remained low for inter-genus, familial, and ordinal relationships regardless of the model of evolution or the partitioning scheme applied to the data (Fig. 1; Table 2), suggesting a lack of phylogenetic information at these levels. Only splits between species of the same genus were strongly and consistently supported (Fig. 1). ML analysis of a Dayhoff-recoded amino acid alignment improved the bootstrap support of internal nodes, particularly for the placement of the root between *Philodina* and the rest of the bdelloids (Fig. 1; Table 2). Parsimony analysis of Dayhoff-recoded sequences recovered the same topology (Fig. 1; Table 2). All 15 most parsimonious trees indicated that *Philodina* was the earliest diverging bdelloid lineage with RHM forming a monophyletic group. In fact, the only variation among these 15 trees was the position of the six *Macrotrachela* sequences with respect to each other.

Depending upon the amino acid substitution model, MrBayes analyses recovered *Philodina* or *Macrotrachela* at the root of the bdelloid tree (Fig. 1.; Table 2). The WAG model supported an RHM clade (as seen in most ML analyses) and *Philodina* as the earliest diverging lineage (Fig. 1., Table 2). In contrast, the MtRev model in MrBayes (selected by the MCMC sampler as the best fitting model under the mixed model parameter) produced a tree with *Macrotrachela* at the root; the same model under ML analysis recovered *Philodina* in this position (Fig. 1, Table 2). Both topologies produced by MrBayes showed strong but conflicting support for internal nodes (Fig. 1; Table 2).

Under a CAT + Poisson model of evolution, Phylobayes recovered the bdelloid root between *Macrotrachela* and the rest of the bdelloids (Fig. 1; Table 2). A combination of GTR (general time reversible) and CAT, which should account for more complexity, recovered *Philodina* at the base of the bdelloid tree, albeit both topologies lack strong support (Fig. 1; Table 2). Essentially, the advantage of employing the CAT model allows equilibrium frequencies to vary among sites. All other analyses in this study employ stationary frequencies –an unrealistic assumption given that some amino acids will tend to replace others more frequently depending on the position. Although the results of Phylobayes are as equally conflicting as ML and MrBayes analyses, the universal themes are that *Macrotrachela* never forms a monophyletic association with *Philodina*. Additionally, in all analyses, the placement of *Habrotrocha* and *Adineta* disrupt the remaining associations among members of Philodinidae and Philodinida (Fig. 1; Table 2).

To determine whether any relationships could be rejected as significantly worse than the traditional schema, I obtained the best ML trees constrained to reflect all possible sister relationships between bdelloid families (Adinetidae + Philodinidae, Adinetidae + Habrotrochidae, and Habrotrochidae + Philodinidae) and evaluated their topologies using the AU and SH tests in CONSEL. No sister relationship could be rejected as implausible using the AU and SH tests, but a sister relationship between Habrotrochidae and Philodinidae was the least likely and on borderline of being rejected (at a significance level of 0.05) according to the AU test (Table 4). The conflicting signal between the likely legitimate relationship of Habrotrochidae and some members of Philodinidae (*Macrotrachela* and *Rotaria*) and the unlikely monophyletic relationship of *Macrotrachela* and or *Habrotrocha* with *Philodina* probably contributes to this result. To test this hypothesis, I also evaluated the plausibility of every possible sister relationship among genera. While the best tree with *Philodina* and *Macrotrachela* as sister taxa falls within the confidence set of trees, it is not as likely as other scenarios (Table 4) and is never observed in my analyses. The AU test rejects a topology with *Philodina* and *Habrotrocha* as sister at a significance level of 0.05 (the SH test fails to reject this relationship but is more conservative Table 4; [56]). This is consistent with the results of ML and Bayesian analyses as *Habrotrocha* never forms a sister relationship with *Philodina* (Fig. 1).

**Table 4:** Bdelloid topology tests.

Relationship tested		lnL	AU	SH	
Habrotrochidae + Adinetidae		−49632	0.77	0.82	
Adinetidae + Philodinidae		−49634	0.32	0.36	
Habrotrochidae + Philodinidae		−49636	0.09	0.21	
*Habrotrocha + Macrotrachela*		−36270	0.74	0.95	
*Adineta* + *Philodina*		−36270	0.58	0.93	
*Rotaria* + *Habrotrocha*		−36275	0.5	0.75	
*Philodina* + *Rotaria*		−36275	0.43	0.72	
*Rotaria* + *Macrotrachela*		−36278	0.29	0.63	
*Philodina* + *Macrotrachela*		−36279	0.36	0.56	
*Adineta* + *Rotaria*		−36282	0.08	0.48	
*Adineta* + *Macrotrachela*		−36288	0.07	0.25	
*Adineta* + *Habrotrocha*		−36293	**0.02**	0.13	
*Philodina* + *Habrotrocha*		−36294	**0.03**	0.13	
**Relationship tested**	**lnL**		**AU**	**SH**	
Lemniscea	−63255, −61722		0.24, 0.98	0.4, 0.99	
Eurotatoria	−63252, −61744		0.49, **0.02**	0.54, **0.02**	
Monogononta + Acanthocephala	−63259, −61745		0.62, **0.01**	0.65, **0.01**	

First three rows represent family relationships tested for Bdelloidea. The remaining rows represent all possible sister relationships among bdelloid genera tested. lnL, log likelihood; AU, P-value for approximately unbiased test; SH, P-value for Shimodaira-Hasegawa test. Boldface indicates statistical significance at the alpha level of <0.05.

### Phylogenetic Analyses of Syndermata

**Figure 2:**
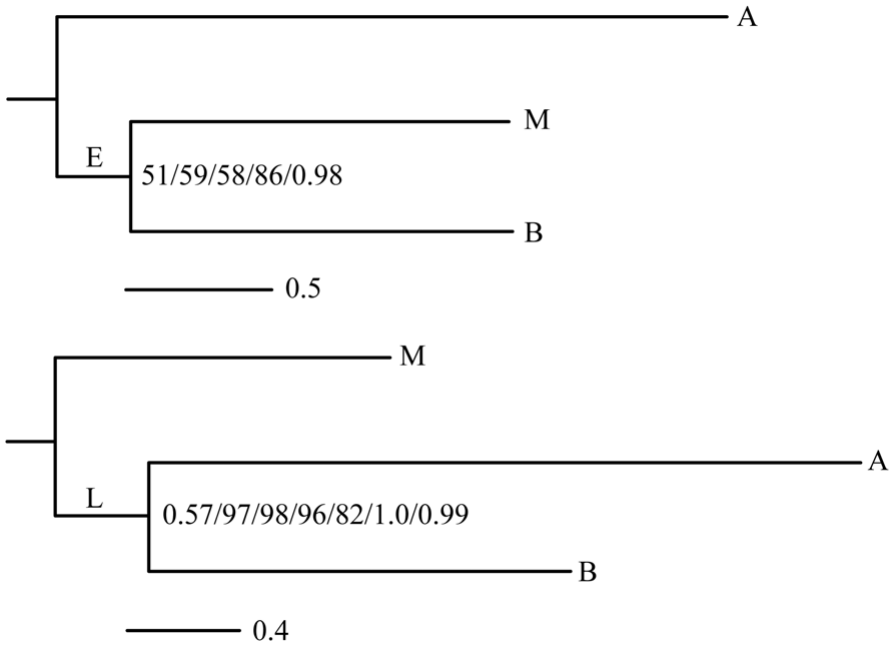
Phylogenies of Syndermata. Syndermate phylogenies reconstructed with ML and Bayesian analyses of concatenated mitochondrial protein alignments and rooted with Platyhelminthes or Chaetognatha. A, Acanthocephala; B, Bdelloidea; M, Monogononta; E, Eurotatoria (Bdelloidea + Monogononta); L, Lemniscea (Bdelloidea + Acanthocephala). Summaries of the two predominant topologies recovered are presented. Only support values for Eurotatoria and Lemniscea are shown as the monophylies of Bdelloidea, Monogononta, and Acanthocephala were always fully supported. All analyses performed with Platyhelminthes, except for one, produced the top phylogeny. Bootstrap support values and posterior probabilities from these analyses are listed in the following order: 1) ML + MtRev, 2) ML + JTT, 3) ML + Partitioned data (models were assigned to each partition and partitions were generated by grouping together genes that shared the same model), 4) ML + JTT on Dayhoff-recoded data, and 5) MrBayes + MtRev. All analyses performed with Chaetognatha and one Platyhelminthes analysis recovered the bottom phylogeny. The first value represents the posterior probability from a Phylobayes analysis of Syndermata + Platyhelminthes under a CAT + Poisson model. The remaining values represent results from Syndermata + Chaetognatha analyses: 1) ML + MtRev, 2) ML + JTT, 3) ML + Partitioned, 4) ML + JTT on Dayhoff-recoded data, 5) MrBayes + MtRev, and 6) Phylobayes with CAT + Poisson model. Lines represent number of amino acid substitutions per site. Summaries of strategies and support values provided in Table 2.

Although lacking a complete representation of Syndermata due to the absence of Seisonidea, it is still informative to compare the relationships of Acanthocephala, Bdelloidea, and Monogononta recovered from my mitochondrial dataset to that of previous mitochondrial analyses. Min and Park consistently recovered a monophyletic Lemniscea in metazoan trees that included *L. thecatus*, *R. rotatoria*, and *B. plicatilis* as representatives of Syndermata but topology tests could not reject the possibility of a monophyletic Eurotatoria [20]. Phylogenies generated from 10 mitochondrial proteins (minus *nd4l* and *atp6*) from *R. rotatoria*, *B. plicatilis*, *L. thecatus*, a second acanthocephalan, *O. luehei*, and other metazoan taxa, recovered the same syndermate relationships as Min and Park with strong bootstrap support and high posterior probabilities [19]. In my ML analyses of a Syndermata + Platyhelminthes dataset, Acanthocephala consistently falls sister to Eurotatoria but bootstrap support is weak (Fig. 2; Table 2). ML analysis of a Dayhoff-recoded alignment dramatically improved the bootstrap support of these relationships (Fig. 2; Table 2) as might be expected when reducing compositional bias or increasing the model violation of the data. MrBayes Bayesian analyses of the same dataset also strongly supported the sister relationship between Bdelloidea and Monogononta (Fig. 2; Table 2) but AU and SH tests could not reject the possibilities of Lemniscea or a sister relationship between Acanthocephala and Monogononta (Table 5). A relative rate test indicated that the mitochondrial sequences of Platyhelminthes are evolving more rapidly than members of Syndermata and that Acanthocephala evolves more rapidly than Bdelloidea or Monogononta, suggesting that the resulting topologies could be influenced by LBA. Implementing the CAT model in Phylobayes to alleviate the potential influence of LBA recovered a phylogeny that weakly supported Lemniscea (Fig. 2; Table 2).

In contrast, rooting Syndermata with Chaetognatha (as represented by *P. gotoi* and *S. cephaloptera*) – a phylum that does not show rapid mitochondrial evolution with respect to Rotifera– consistently recovered Lemniscea with strong bootstrap support, regardless of amino acid model, partitioning, or Dayhoff-recoding for ML analyses (Fig. 2; Table 2). All Bayesian analyses also strongly supported Lemniscea, providing total congruency among all models and methods of inference (Fig. 2; Table 2). The AU and SH tests soundly rejected Eurotatoria and a sister relationship between Acanthocephala and Monogononta when rooting the three possible syndermate topologies with Chaetognatha (Table 5).

**Figure 3:**
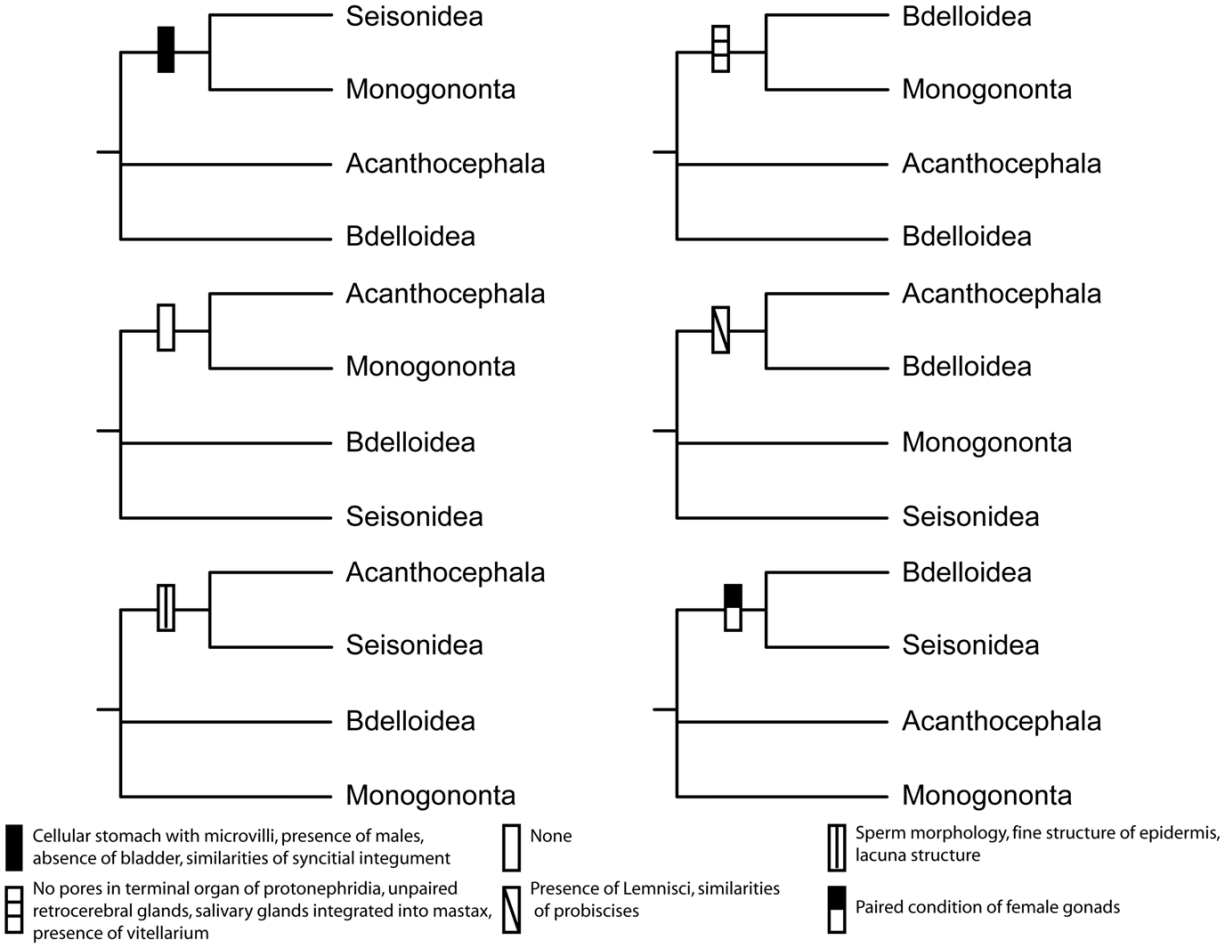
Possible sister relationships among lineages of Syndermata and the morphological support for each.

**Table 5:** Syndermate topology tests.

Relationship tested	lnL	AU	SH
Lemniscea	−63255, −61722	0.24, 0.98	0.4, 0.99
Eurotatoria	−63252, −61744	0.49, **0.02**	0.54, **0.02**
Monogononta+Acanthocephala	−63259, −61745	0.62, **0.01**	0.65, **0.01**

The two values for each entry represent the topology tests conducted with Platyhelminthes (first value) and Chaetognatha (second value) as outgroups.Relationships tested included Lemniscea (Bdelloidea+Acanthocephala), Eurotatoria (Bdelloidea+Monogononta), and Monogononta+Acanthocephala.lnL, log likelihood; AU, P-value for approximately unbiased test; SH, P-value for Shimodaira-Hasegawa test.Boldface indicates statistical significance at the alpha level of <0.05.

## Discussion

My results indicate that neither Philodinidae nor Philodinida represent phylogenetically informative entities. The placement of *Philodina* or *Macrotrachela* at the base of the bdelloid tree single-handedly disrupts both classifications. The sister relationship frequently recovered between *Habrotrocha* and *Macrotrachela* also scrambles the Philodinidae family. However, the inconsistent results produced despite attempting to account for compositional biases and substitution rate saturation indicates that these mitochondrial sequences do not contain enough phylogenetic information for greater insight into bdelloid relationships. Better methods of analysis to address the problems created by short internal branch lengths, compositional biases, and potential artifacts generated by the long branch between outgroup and ingroup are also necessary to increase resolving power. Recoding amino acid alignments into Dayhoff categories might be a cost-effective alternative to amassing more sequences for phylogenetic resolution but the loss of information incurred from recoding is an undesirable solution when dealing with a possible rapid radiation of bdelloid lineages. Site-heterogeneous models such, as CAT, in combination with methods that assign different substitution models to different lineages (non-stationary models) appear to be the best solutions for uncovering relationships among problematic taxa but at the expense of computational time. Increased taxonomic sampling, particularly from members of the missing family, Philodinavidae might enhance the resolution of the bdelloid tree. Although my dataset improves the taxonomic representation of bdelloids and the sample size of monogononts, all three monogononts represent the same order. Sampling other monogonont orders might provide a representative of this class that does not fail the test of compositional homogeneity. A less rapidly evolving member of Acanthocephala might also provide more topological stability and break up the long branch from outgroup (Monogononta + Acanthocephala) to ingroup (Bdelloidea). In the end, the ambiguous nature of the reconstructed bdelloid phylogenies suggests that close associations among members of different families and orders might be as equally probably as traditional ideas regarding bdelloid evolution. Members of Bdelloidea display unusual characteristics such as the ability to survive desiccation at any life stage [57] and extreme resistance to ionizing radiation [58], which have made them candidates for aging research (Meselson, Personal communication). Yet, little attention has been given to disentangling the bdelloid phylogeny, particularly at the molecular level [9], [10]. Any study promoting bdelloids as model systems would greatly benefit from putting these traits into an evolutionary framework. In general, the classification of these small invertebrates that are often difficult to examine morphologically would greatly benefit from an accurate molecular-based phylogeny.

Current morphological and molecular data are inconclusive about the relationships among lineages of Syndermata. From a morphological perspective, Rotifera, *sensu strictu* (Monogononta, Bdelloidea, and Seisonidea), share more obvious synapomorphies with each other, such as coronas and trophi (jaw structures), than the highly modified Acanthocephala. However, relationships between Rotifera and Acanthocephala based on strict morphology are difficult to establish due to the lack of characters available for comparison, particularly since acanthocephalans are highly modified for a parasitic lifestyle [59]. While the presence of a syncytial epidermis appears to unite Rotifera and Acanthocephala [45], other morphological characteristics propose conflicting scenarios for the placement of Acanthocephala with respect to the three classes of Rotifera and for relationships among Rotifera. The lemnisci (sac-like structures involved in metabolism) and proboscis (protrudable feature that pierces the host) of Acanthocephala and the sac-like organs and rostrum (an adhesive structure that aids in creeping movement with the foot) in Bdelloidea either support a sister relationship between these two groups [60] or contribute to placing Acanthocephala as sister to Rotifera [8], [61] depending on the interpretation of these structures as homologous (Fig. 3). Within Rotifera, analyses based primarily on trophi and sperm morphology support a sister relationship between Eurotatoria [52], [62] (Fig. 3). Other characters considered synapomorphic for Eurotatoria are: the absence of pores in the terminal organ of the protonephridia, unpaired retrocerebral glands, salivary glands integrated into the mastax, and the presence of a vitellarium [63], [64] (Fig. 3). Seisonidea falls sister to: 1) Bdelloidea based on the condition of paired female gonads [65] or 2) Monogononta based on the presence of males, a cellular stomach with microvilli, shared features in the syncytial integument, and the absence of a bladder [3] or 3) Acanthocephala based on the ultrastructural analysis of spermatozoa [45] (Fig. 3). In other words, morphological features recover every possible sister relationship among the four major clades of Syndermata except for a monogonont/acanthocephalan group.

Molecular analyses thus far support a monophyletic Syndermata but fail to recover consistent relationships among syndermate lineages [5], [50], [59], [66], [67], [68]. The gene(s) comprising each dataset and the different analytical methods and/or taxonomic sampling strategies employed all contribute to different results. For example, the small ribosomal subunit (*ssu* rDNA) recovers a sister relationship between Lemniscea in the absence of Seisonidea but recovers a well-supported relationship between Seisonidea and Acanthocephala when Seisonidea is present [50], [66], [68]. A phylogenomic analysis of 79 ribosomal proteins recovers Lemniscea but does not include a representative of Seisonidea [69]. The nuclear-encoded heat shock protein, *hsp82*, supports a sister relationship between Acanthocephala and Eurotatoria, with Seisonidea as the earliest diverging syndermate lineage but significance tests could not reject alternative topologies [70]. A combined dataset of *hsp82* and *ssu* rDNA also supports Eurotatoria but maintains Acanthocephala within Rotifera as either sister to Eurotatoria or Seisonidea [71]. In contrast, combined analyses of *ssu* and *lsu* rDNA, and mitochondrial *cox1* consistently recover Lemniscea but the position of Seisonidea or Monogononta as sister to this clade changes depending on the gene combination and method of phylogenetic inference [72]. Another multigene analysis (*cox1*, histone H3, *lsu*, and *ssu* rDNA) placed Seisonidea with Acanthocephala or Bdelloidea depending on the method of inference [59].

Two previous mitogenomic analyses recover Lemniscea but Seisonidea was not represented and Bdelloidea and Monogononta were only represented by *R. rotatoria* and *B. plicatilis*, respectively [19], [20]. A phylogenetic analysis performed with over 1000 *cox1* sequences from all members of Syndermata suggests that increasing taxonomic representation changes the topology recovered as both ML and Bayesian phylogenies of this dataset supported Eurotatoria [9]. However, SH tests indicated that topologies with a monophyletic Lemniscea were most likely [9]. From these three studies, it is unclear whether increasing characters or increasing taxonomic sampling provided any benefit in resolving syndermate relationships.

The inclusion of an outgroup contributes the strongest topological influence in my analyses. Lemniscea appears to be a legitimate clade pulled apart by the artificial attraction of Acanthocephala to Platyhelminthes as both lineages are rapidly evolving. Although bootstrap values and posterior probabilities are not equivalent measures, such large differences between the bootstrap support values of my ML trees and the posterior probabilities of my Bayesian trees for the Syndermata + Platyhelminthes dataset are reminiscent of the effects of LBA on simulated data [73]. Dayhoff-recoding improves bootstrap support for Eurotatoria in the Syndermata + Platyhelminthes tree but employing the CAT model - a more sophisticated method of inference – recovers Lemniscea instead. The inability of Dayhoff-recoding to recover Lemniscea is not surprising given that all taxa except the bdelloids still fail the test for compositional homogeneity. Similarly, all empirical models employed by MrBayes and RAxML suffer from the same problem – the assumption of a site homogeneous substitution process. In other words, all RAxML and MrBayes analyses are subject to same systematic bias and result in the same syndermate topology. Further support for Lemniscea stems from replacing Platyhelminthes with Chaetognatha - a less rapidly evolving outgroup. Regardless of data manipulation or model complexity, all strategies arrive at the same answer – a well-supported Lemniscea. Thus, my results highlight the importance of not only applying different methods of inference but also modifying the taxonomic representation of one’s dataset. Without reconstructing the syndermate phylogeny with Phylobayes or a Chaetognatha outgroup, majority rule would favor a most likely incorrect sister relationship between Bdelloidea and Monogononta. Ultimately, key factors to confirming the monophyly of Lemniscea will be to obtain mitochondrial sequences from the missing rotifer class Seisonidea and to obtain sequences from a more closely related outgroup (such as Gnathostomulida) that reduces the potential for LBA. Resolving the phylogeny of Syndermata not only represents a unique opportunity to study the evolution of asexuality, parasitism, and commensalism within a single lineage and to examine the genomic modifications that occur in association with each lifestyle and mode of reproduction but resolving the phylogeny of Syndermata also contributes to our greater understanding of metazoan evolution in general.

## Supporting Information

Table S1 **ProtTest Results.**
(DOCX)
