# Supplementary material for: A Mitogenomic Re-Evaluation of the Bdelloid Phylogeny and Relationships among the Syndermata
Source: PLoS One. 2012 Aug 23;7(8):e43554. doi: 10.1371/journal.pone.0043554 (PMC3426538; doi:10.1371/journal.pone.0043554)
Supplement: Table S1 — ProtTest Results. (DOCX) [file pone.0043554.s001.docx]

| **Table S1.** ProtTest Results. Amino acid substitution model assigned to each dataset. S, syndermate only alignment; SP, syndermate + platyhelminthes alignment; SC, syndermate + chaetognath alignment. +I, proportion of invariant sites +G, gamma-distribution; +F, amino acid frequencies from data | | | | | | | | | | | | |
| --- | --- | --- | --- | --- | --- | --- | --- | --- | --- | --- | --- | --- |
| **Data** | ***Atp6*** | ***Cox1*** | ***Cox2*** | ***Cox3*** | ***Cytb*** | ***Nd1*** | ***Nd2*** | ***Nd3*** | ***Nd4*** | ***Nd4l*** | ***Nd5*** | ***Nd6*** |
| S | JTT+G+F | MtArt+G+F | MtArt+G+F | MtREV+G+F | JTT+G+F | MtMam+G+F | JTT+I+G+F | MtRev+G+F | JTT+G+F | JTT+G+F | JTT+I+G+F | MtREV+G+F |
| SP | JTT+G+F | MtArt+G+F | JTT+I+G+F | MtArt+G+F | JTT+G+F | MtArt+G+F | JTT+G+F | LG+G+F | JTT+G+F | JTT+G+F | JTT+G+F | MtREV+G+F |
| SC | JTT+G+F | JTT+I+G+F | MtArt+G+F | JTT+G+F | JTT+G+F | MtArt+G+F | JTT+I+G+F | MtRev+G+F | JTT+I+G+F | JTT+G+F | JTT+I+G+F | MtREV+I+G+F |
